# Multicenter Case–Control Study of Behavioral, Environmental, and Geographic Risk Factors for Talaromycosis, Vietnam 

**DOI:** 10.3201/eid3107.250143

**Published:** 2025-07

**Authors:** Lottie Brown, Brian Jonat, Vo Trieu Ly, Nguyen Le Nhu Tung, Pham Si Lam, Nguyen Tat Thanh, Dang Thi Ngoc Bich, Vu Phuong Thao, Nguyen Thi Mai Thu, Ngo Thi Hoa, Thuy Le

**Affiliations:** City St. George’s Hospital and St. George’s University, London, UK (L. Brown); Boehringer Ingelheim Pharmaceuticals, Inc., Ridgefield, Connecticut, USA (B. Jonat); Tropical Medicine Research Center for Talaromycosis, Pham Ngoc Thach University of Medicine, Ho Chi Minh City, Vietnam (V.T. Ly, N.T. Hoa, T. Le); Hospital for Tropical Diseases, Ho Chi Minh City (V.T. Ly, N.L.N. Tung); Oxford University Clinical Research Unit, Ho Chi Minh City (P.S. Lam, N.T. Thanh, N.T. Hoa); National Hospital for Tropical Diseases, Hanoi, Vietnam (D.T.N. Bich); Saskatchewan Cancer Agency, Regina, Saskatchewan, Canada (V.P. Thao); Duke University Medical Center, Durham, North Carolina, USA (N.T.M. Thu, T. Le)

**Keywords:** talaromycosis, fungi, bamboo rat, HIV/AIDS and other retroviruses, penicilliosis, *Penicillium marneffei*, risk factors, *Talaromyces marneffei*, Vietnam

## Abstract

Talaromycosis is a life-threatening fungal disease that primarily affects immunocompromised persons in Southeast Asia. We conducted a multicenter, case–control study recruiting participants with advanced HIV disease in Vietnam; 205 case-patients with culture-confirmed talaromycosis were matched to 405 control-patients by age, sex, and CD4 count. Occupational exposure to tropical plants (odds ratio [OR] 1.73 [95% CI 1.10–2.73]; p = 0.017) and to farmed animals (OR 2.07 [95% CI 1.20–3.55]; p = 0.009) were independent risk factors for talaromycosis. Talaromycosis risk was higher in participants from highland regions than in persons from lowland regions (p<0.05). Participants from lowland regions who had lived or traveled to highland regions had a higher risk for talaromycosis (OR 3.15 [95% CI 1.49–6.64]; p = 0.003). This study confirms the epidemiologic correlation between talaromycosis and soil exposure and demonstrates an epidemiologic link between talaromycosis and residence in or travel to highland regions of Vietnam.

Talaromycosis (formerly penicilliosis) is an invasive fungal disease caused by *Talaromyces marneffei*, a dimorphic fungus endemic to Southeast Asia. First discovered in captive wild bamboo rats (*Rhizomys sinensis*) in Vietnam in 1956, talaromycosis remained rare in humans until the onset of the HIV epidemic in the 1980s, when it rapidly emerged as a leading HIV-associated opportunistic infection and cause of HIV-associated death in Southeast Asia (*1*,*2*). The highest reported disease burden is in northern Thailand, Vietnam, and southern China, where talaromycosis accounts for 4%–20% of HIV-related hospital admissions and the mortality rate, despite antifungal treatment, is between 15% and 30% (*2*–*7*). Outside of those hyperendemic regions, talaromycosis is likely underdiagnosed, and the true burden of disease across Southeast Asia remains unknown (*7*,*8*). Although most cases (≈90%) are associated with advanced HIV disease, the incidence of talaromycosis is rising among persons with other immunocompromising conditions, such as primary immunodeficiency, malignancy, solid organ or stem cell transplant, and autoimmune or inflammatory conditions that require immunomodulating therapy (*9*–*11*). Cases are increasingly reported outside of talaromycosis-endemic regions because of increased migration and international travel (*8*).

A lack of understanding of disease reservoirs and exposure risk factors for talaromycosis has hampered disease prevention. The bamboo rat is the only known enzootic reservoir of *T. marneffei*. Surveys of rodents in talaromycosis-endemic regions have identified all 4 species of bamboo rat in Southeast Asia (*R. sinensis, R. pruinosis, R. sumatrensis,* and *Cannomys badius*) (Appendix Figure 1) as asymptomatic carriers of *T. marneffei*; reported prevalence ranges from 10% to 100% (*12*–*15*). The geographic distribution of bamboo rats follows the distribution of human talaromycosis (Appendix Figure 2), and *T. marneffei* isolates from bamboo rats have been shown to share similar genotypes to those infecting humans in the same region (*16*,*17*), suggesting the potential for bamboo rat–to–human transmission. However, epidemiologic evidence of direct bamboo rat–to–human transmission is lacking. A case–control study conducted in Chiang Mai, a rural and mountainous region of Thailand where bamboo rats are endemic, demonstrated no link between exposure or consumption of bamboo rats and human talaromycosis. Instead, agricultural work and soil exposure, particularly during the rainy season, were independent risk factors for talaromycosis (*18*). Because *T. marneffei* has been isolated more frequently in soil samples collected from bamboo rat burrows than from non–bamboo rat habitats (*16*,*19*), we hypothesize that exposure to soil associated with bamboo rats could be the primary driving factor for talaromycosis, rather than direct exposure to bamboo rats.

Aside from soil exposure, weather factors have a substantial effect on talaromycosis incidence. During the monsoon season, the incidence of talaromycosis rises 30% in Vietnam, 50% in Thailand, and 73% in southern China from that observed during the dry season and has been associated with increased temperatures and humidity, which provide favorable environmental conditions for *T. marneffei* (*4*,*6*,*20*,*21*). Talaromycosis is also known to occur in geographic hot spots in mountainous areas of Southeast Asia, such as the northern provinces of Chiang Mai and Chiang Rai in Thailand, the southern provinces of Guangdong and Guangxi in China, and the northeastern states of Manipur and Assam in India (*22*). Vietnam presents a unique opportunity to study the geographic and exposure risk factors for talaromycosis because talaromycosis occurs across diverse climatic and geographic ranges, from the temperate, subtropical regions in the north to the tropical monsoon regions in the south (Figure, panel A). Three quarters of the land in Vietnam is composed of highlands (defined as hills and mountains >400 m above sea level), which include the Northeast and Northwest regions in the north and the Central Highland region in the south. The remaining one quarter of the land consists of lowlands, which include the Red River Delta in the north, the coastline, and the Mekong Delta in the south. Talaromycosis is seen in both rural and urban settings in Vietnam, where risk factors for infection might be very different than in the rural settings of Chiang Mai, Thailand (*18*). This study aimed to evaluate the behavioral, environmental, and geographic risk factors for talaromycosis in the diverse climatic and geographic setting of the hyperendemic country of Vietnam.

**Figure:**
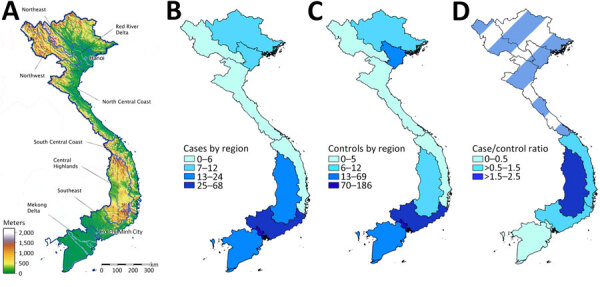
Geographic distribution of recruited cases and controls in multicenter study of behavioral, environmental, and geographic risk factors for talaromycosis, Vietnam. A) Municipal regions of Vietnam, showing topography and locations of recruitment centers in Hanoi and Ho Chi Minh City; B) number of cases by region; C) number of controls by region; D) ratio of cases to controls by region. Darker color represents a higher number of cases relative to controls for that region. A case-to-control ratio of 0.5 is expected because 2 controls were recruited for every case. A ratio <0.5 indicates a lower-than-expected number of cases relative to controls. A ratio >0.5 indicates a greater-than-expected number of cases relative to controls. Data for northern regions of Vietnam are excluded from panel D because study enrollment from these regions was insufficient to draw meaningful conclusions from the analysis. Both cases and controls are concentrated in Southern Vietnam, where most participants were recruited. The ratios of cases to controls are higher in the Central Highlands and the adjacent Southeast and South-Central Coast regions than in Ho Chi Minh City and the Mekong Delta; the highest ratio was seen in the Central Highlands region.

## Methods

### Study Design and Participants

We conducted a multicenter, matched, case–control study recruiting participants with advanced HIV disease from the inpatient and outpatient departments of the National Hospital for Tropical Diseases (Hanoi, Vietnam) and the Hospital for Tropical Diseases (Ho Chi Minh City, Vietnam), during January 2013–July 2016. Those hospitals were estimated to receive 40% of all HIV inpatient referrals in Vietnam, which has a catchment population of 72 million. We predetermined risk factors of interest (n = 13) after a comprehensive literature review and included behavioral, environmental, and geographic risk factors (Table 1).

**Table 1:** Thirteen predefined exposure variables in multicenter case–control study of behavioral, environmental, and geographic risk factors for talaromycosis, Vietnam

Exposure variable	Definition
1. Antiretroviral therapy	Current use of antiretroviral therapy
2. Antifungal prophylaxis	Current use of antifungal prophylaxis
3. Cigarette smoking	Current cigarette smoking
4. Injection drug use	Current injection drug use
5. Outdoor occupations	Current or previous job in construction, agriculture, gardening, or long-distance truck driving
6. Soil exposure	Living within 100 meters of agricultural/industrial soil-excavation sites or direct occupational exposure to construction work, farming, soil digging, gardening, or rubbish collection
7. Natural water	Living within 100 meters of or direct occupational exposure to river, lake, pond, canal, or ditch
8. Tropical plants	Living within 100 meters of or direct occupational exposure to any type of bamboo, sugar cane, or rice
9. Highland plants	Living within 100 meters of or direct occupational exposure to rubber, cashew, coffee, or tea
10. Bamboo rats	Any direct contact or consumption with bamboo rats
11. Farming animals	Direct occupational exposure to pigs, cows, chickens, or ducks
12. Domestic animals	Frequent contact or caring of dogs, cats, birds, or fishes
13. Raw animal products	Any consumption of uncooked meats, eggs, milk, or blood

We consecutively recruited participants from a population of adults with HIV >18 years of age with a CD4 cell count <100 cells/µL who had culture-proven talaromycosis (defined as a compatible clinical syndrome and isolation of *T. marneffei* in blood, skin lesion, bone marrow, lymph node, or other specimens as clinically indicated). We consecutively recruited control-patients who came from the same at-risk population but had no clinical suspicion for or microbiological evidence of talaromycosis. To reduce the potential confounding effect, we matched cases to controls at a 1:2 ratio according to age (+5 years), sex, and CD4 cell count +50 cells/µL. When a CD4 count was not performed or available, we matched participants by absolute lymphocyte count (+330 cells/µL, calculated on the basis of an assumed 15% CD4-positive lymphocyte count, 50/0.15 = 330) or HIV disease at stage 3 or 4 according to World Health Organization (WHO) clinical staging. We recruited control-patients within the same week as the corresponding case-patients from the same site.

### Sample Size Estimation

We calculated the sample size estimate for the matched case–control study according to Parker and Bregman (*23*). The target sample size of 200 in the case group and 400 in the control group was chosen to allow 90% power to detect an odds ratio (OR) of >1.9 for a risk factor, assuming a proportion of risk exposure of 0.2.

### Data Collection

After participant recruitment, trained study nurses conducted face-to-face interviews using a standardized questionnaire of prespecified exposure variables (Table 1). To control for assessment bias, study nurses were blinded to the assignment of case and control and the study hypotheses. A photograph of the 4 species of bamboo rats (Appendix Figure 1) was shown to all participants to ensure that the history of exposure was correctly elicited. We assigned participants to 8 municipal regions based on current residential address. We categorized data from Ho Chi Minh City as a separate region because of the high number of participants recruited within the city limits. We performed a posthoc follow-up of participants residing in lowland regions (Ho Chi Minh City and the Mekong Delta) to investigate the risk posed by previous travel to or previous residence in highland regions that had been identified through the geographic mapping to be talaromycosis risk regions. The posthoc follow-up consisted of phone interviews conducted by research nurses who were blinded to the participant status as case or control.

### Statistical Analysis

We used univariate and multivariable conditional logistic regressions to evaluate risk factors for talaromycosis using a complete case analysis. We included all 13 predefined exposure variables (Table 1) in the regression model. The Central Highlands and some provinces in the adjacent Southeast and South Central Coast regions in southern Vietnam consist of hills and mountainous terrain >400 meters in elevation (Figure, panel A). To investigate whether participants residing in those highland regions were at greater risk for talaromycosis than were residents of the lowland regions of Ho Chi Minh City and the Mekong Delta, we performed conditional logistic regression with the region as the only covariate. We adjusted pairwise comparisons between regions for multiple comparisons using a parametric single-step method (*24*). Participants recruited in northern Vietnam were excluded from this geographic analysis because of the later start date, which meant a substantially smaller number of patients were recruited with too diverse geographic distribution to enable robust statistical analysis in northern regions. We estimated the number of talaromycosis cases recruited per total HIV population in each region using HIV prevalence data obtained from the Vietnam Ministry of Health Administration of HIV/AIDS Control from 2007 to evaluate for any bias in referral pattern (*25*).

We visualized maps of the number of cases, controls, and case-to-control ratios in different geographic regions in southern Vietnam using the Quantum Geographic Information System version 1.5 (https://qgis.org). We performed all statistical analyses by using R version 3.2.1 (The R Project for Statistical Computing, https://www.r-project.org).

### Ethics

This case–control study was a substudy of the Itraconazole and Amphotericin B for Talaromycosis clinical trial that was approved by the Vietnam Ministry of Health (protocol 781-Vietnam MOH), by the Oxford University Tropical Research Ethics Committee (protocol OxTREC 12–09), and by the ethical and scientific committees of the 2 study sites in Vietnam. All participants provided written consent before study enrollment.

## Results

### Characteristics of Study Participants

During January 2013–July 2016, a total of 236 cases of talaromycosis were diagnosed across the 2 centers, of which 205 cases were recruited into the study and matched to 405 controls. Of the 31 cases screened but not recruited (5.1%), 25 died or were discharged before the talaromycosis diagnosis was confirmed (80.6%), and the remaining 6 declined to participate. Participants were mostly men (75% [456/610]), consistent with the HIV-infected population in Vietnam. Median age was 34 years (interquartile range [IQR] 31–38 years), and median CD4 count was 17 cells/μL (IQR 7–36). Case-patients and control-patients were similar in age, sex, and absolute lymphocyte counts (Table 2). CD4 count was significantly lower in case-patients than in control-patients. According to WHO HIV disease staging, all case-patients were classified as stage 4 (severe), whereas control-patients were mostly stage 3 or 4 (moderate or severe). Most participants were inpatient (100% [205/205] of case-patients and 91% [369/405] of control-patients). Other opportunistic infections, most commonly tuberculosis, pneumonia (caused by bacteria or *Pneumocystis*), oral/esophageal candidiasis, or cryptococcosis, were diagnosed in controls. Concurrent opportunistic infections were common among case-patients with talaromycosis (Table 2).

**Table 2:** Characteristics of participants in multicenter case–control study of behavioral, environmental, and geographic risk factors for talaromycosis, Vietnam*

Characteristic	All patients, N = 610	Cases, n = 205	Controls, n = 405	p value†
Median age, y (IQR)	34 (31–38)	33 (30–38)	34 (31–39)	0.401
Sex				
M	456 (74.8)	154 (75.1)	302 (74.6)	0.882
F	154 (25.2)	51 (24.9)	103 (25.4)	
Median CD4, cells/µL (IQR)	16.5 (7.0–36.0), n = 194	9.0 (5.0–18.8), n = 66	25.5 (9.0–54.3), n = 128	0.003
Median absolute lymphocyte, cells/µL (IQR)	520 (300–750), n = 585	410 (230–600), n = 197	570 (380–810), n = 388	0.184
WHO stage	n = 606	n = 204	n = 402	
1	3 (0.5)	0	3 (0.7)‡	0.217
2	16 (2.6)	0	16 (4.0)‡	0.004
3	146 (24.1)	0	146 (36.3)‡	<0.001
4	441 (72.8)	204 (100)	237 (59.0)	<0.001
Hospitalization status				
Inpatient	573 (93.9)	205 (100)	368 (90.9)	<0.001
Outpatient	37 (6.1)	0	37 (9.1)	
Concomitant opportunistic infections				
Nontuberculosis pneumonia, including PcP	130 (21.3)	24 (11.7)	106 (26.2)	<0.001
Oral/esophageal candidiasis	90 (14.8)	14 (6.8)	76 (18.8)	<0.001
Tuberculosis	84 (13.8)	23 (11.2)	61 (15.1)	0.193
Cryptococcosis	45 (7.4)	0	45 (11.1)	<0.001
Toxoplasmosis	40 (6.6)	1 (0.5)	39 (9.6)	<0.001
Herpes simplex virus	12 (2.0)	3 (1.5)	9 (2.2)	0.524
AIDS-associated wasting syndrome	23 (3.8)	2 (1.0)	21 (5.2)	0.010
Other opportunistic infection	73 (12.0)	22 (10.7)	51 (12.6)	0.504
No opportunistic infection	66 (10.9)	0	66 (16.3)	<0.001

*Values are no. (%) except as indicated. IQR, interquartile range; PcP, *Pneumocystis* pneumonia; WHO, World Health Organization. †χ^2^ test was used for categorical variables and 2-tailed Student *t*-test was used for continuous variables. ‡These control patients had CD4 counts or absolute lymphocyte counts in the same ranges as their matched case-patients.

### Analysis of Behavior and Environmental Risk Factors

Data were 100% complete for all 610 participants for 12 of 13 covariables (Table 1). Data were incomplete for only 1 area, fluconazole prophylaxis, in which data were missing for 14 (2%) participants (Table 3). In the univariate analysis, patients with talaromycosis were significantly more likely to work in outdoor settings, live within 100 meters of or have direct occupational contact with a tropical plant (bamboo, sugar cane, or rice), or have direct occupational contact with a highland plant (rubber, tea, coffee) and farm animals (cattle, swine, poultry). In the multivariable analysis, exposure to tropical plants (OR 1.73 [95% CI 1.10–2.73]; p = 0.017) and exposure to farm animals (OR 2.07 [95% CI 1.20–3.55]; p = 0.009) were the only independent risk factors for talaromycosis (Table 3).

**Table 3:** Univariate and multivariable conditional logistic regression analysis of risk factors for talaromycosis in multicenter case–control study of behavioral, environmental, and geographic risk factors for talaromycosis, Vietnam*

Exposure covariate	All patients, N = 610	Cases, n = 205	Controls, n = 405	Univariate effect			Multivariate effect	
				OR (95% CI)	p value		OR (95% CI)	p value
Antiretroviral therapy	250/610 (41.0)	72/205 (35.1)	178/405 (44.0)	**0.68 (0.47–0.97)**	**0.04**		0.75 (0.50–1.13)	0.17
Fluconazole prophylaxis	61/596 (10.2)	15/198 (7.6)	46/398 (11.6)	0.59 (0.31–1.11)	0.10		0.68 (0.35–1.34)	0.27
Cigarette smoking	413/610 (67.7)	130/205 (63.4)	283/405 (69.9)	0.65 (0.42–1.01)	0.06		0.71 (0.43–1.18)	0.19
Injection drug use	232/610 (38.0)	71/205 (34.6)	161/405 (39.8)	0.79 (0.54–1.15)	0.21		0.85 (0.54–1.35)	0.50
Outdoor occupation	263/610 (43.1)	100/205 (48.8)	163/405 (40.2)	**1.47 (1.03–2.09)**	**0.04**		1.23 (0.81–1.87)	0.34
Soil exposure	409/610 (67.0)	143/205 (69.8)	266/405 (65.7)	1.22 (0.85–1.75)	0.29		1.06 (0.69–1.63)	0.80
Natural water exposure	285/610 (46.7)	90/205 (43.9)	195/405 (48.1)	0.83 (0.58–1.19)	0.31		0.76 (0.51–1.13)	0.18
Tropical plant exposure	218/610 (35.7)	90/205 (43.9)	128/405 (31.6)	**1.75 (1.22–2.56)**	**0.002**		**1.84 (1.17–2.90)**	**0.008**
Highland plant exposure	49/610 (8.0)	25/205 (12.2)	24/405 (5.9)	**2.25 (1.24–4.01)**	**0.008**		1.71 (0.86–3.41)	0.13
Bamboo rat exposure or consumption	6/610 (1.0)	3/205 (1.5)	3/405 (0.7)	2.00 (0.40–9.91)	0.40		1.71 (0.33–8.87)	0.53
Farming animal exposure	93/610 (15.2)	40/205 (19.5)	53/405 (13.1)	**1.60 (1.02–2.51)**	**0.04**		**2.03 (1.18–3.49)**	**0.010**
Domestic animal exposure	170/610 (27.9)	57/205 (27.8)	113/405 (27.9)	1.01 (0.67–1.51)	0.97		1.39 (0.87–2.22)	0.17
Raw animal product consumption	411/610 (67.4)	132/205 (64.4)	279/405 (68.9)	0.82 (0.57–1.18)	0.28		0.91 (0.60–1.37)	0.64

*Values are no. (%) except as indicated. OR is based on conditional logistic regression. Multivariate analysis is a complete case analysis excluding 7 cases and 7 controls with missing fluconazole prophylaxis data. A p value <0.05 is considered statistically significant and highlighted in bold. For definitions of exposure covariates, refer to Table 1. OR, odds ratio.

### Mapping of Cases and Controls and Geographic Risk Analysis

Geographic data were available for 204 (99.5%) of 205 case-patients and 403 (99.5%) of 405 control-patients (Figure, panels B, C). We performed mapping and geographic analysis only for southern Vietnam because recruitment in southern Vietnam began earlier and more case-patients (86% [175/205]) and control-patients (86% [348/405]) were enrolled in southern Vietnam to provide robust data for these analyses. The case-to-control ratio demonstrated regions with a higher or lower than expected number of cases relative to controls; 0.5 was the expected case-to-control ratio, given that 2 controls were recruited for each case (Figure, panel D). We found that case-to-control ratios were highest in the Central Highlands (2.18), followed by the surrounding provinces in the South-Central Coast (1.20) and the Southeast region (0.87). Case-to-control ratios were the lowest in Ho Chi Minh City (0.32) and the Mekong Delta (0.25), where the number of persons recruited was the highest (Figure, panel D). Conditional logistic regression demonstrated that participants in the highland regions in the Central Highlands, Southeast, and South-Central Coast were significantly more likely to develop talaromycosis than those residing in the lowlands of Ho Chi Minh City and Mekong Delta (multiplicity-corrected p<0.05 for all pairwise comparisons) (Table 4). The point estimate of the risk for talaromycosis was higher in the Central Highlands than in the Southeast lowland region, suggesting a risk exposure relationship for closer proximity to the highest risk Central Highland region, but this difference did not reach statistical significance (OR 3.32; p = 0.06). In the southern regions of Vietnam, the highest relative number of talaromycosis cases was in the Central Highlands (Table 5).

**Table 4:** Case-to-control ratio and risk for talaromycosis per region in multicenter case–control study of behavioral, environmental, and geographic risk factors for talaromycosis, Vietnam*

Region†	No. cases	No. controls	Case-to-control ratio	OR (95% CI)‡	p value
Mekong Delta	17	69	0.25	Referent	
HCMC	60	185	0.32	1.31 (0.56–3.03)	0.91
Southeast	68	78	0.87	3.42 (1.44–8.10)	0.001
South Central Coast	6	5	1.20	8.76 (1.25–61.56)	0.02
Central Highlands	24	11	2.18	11.36 (2.92–44.24)	<0.0001
Total	175	348	0.5		

*Conditional logistic regression analysis with the region as the only covariate was conducted to investigate whether participants residing in the highland regions were at greater risk for talaromycosis than were residents of the lowland regions of HCMC and the Mekong Delta. Pairwise comparisons between regions were adjusted for multiple comparisons using a parametric single-step method. HCMC, Ho Chi Minh City; OR, odds ratio. †Participants recruited in northern Vietnam were excluded from this geographic analysis because of the later start date and, hence, a smaller number of patients in northern regions were recruited. ‡OR is based on conditional logistic regression. p values are multiplicity-adjusted for pair-wise comparisons between all regions. Comparisons to the Mekong Delta are shown above. The remaining pair-wise comparisons are comparisons to HCMC: Southeast OR 2.62 (1.41–4.85); p = 0.0003; South Central Coast OR 6.71 (1.04–43.45); p = 0.04; Central Highlands OR 8.70 (2.61–28.97); p<0.0001. Comparisons to Southeast: South Central Coast OR 2.57 (0.40–16.52); p = 0.63; Central Highlands OR 3.32 (0.98–11.26); p = 0.06. Comparison to Central Highlands: South Central Coast OR 0.77 (0.12 – 5.06); p = 1.00.

**Table 5:** Recruited talaromycosis cases per regional HIV population in southern Vietnam in multicenter case–control study of behavioral, environmental, and geographic risk factors for talaromycosis, Vietnam*

Region	Talaromycosis cases	Estimated HIV population, 2007	Cases/100,000 HIV population
Mekong Delta	17	103,615	16
Ho Chi Minh City	60	72,566	83
Southeast	68	52,132	130
South Central Coast	6	11,878	51
Central Highlands	24	12,123	200

*The number of talaromycosis cases recruited per total HIV population in each region was estimated using HIV prevalence data obtained from the Vietnam Ministry of Health Administration of HIV/AIDS Control from 2007 to evaluate for any bias in referral pattern.

To investigate whether previous travel to or residence in any of the 3 identified risk regions was associated with the risk for talaromycosis, we performed a posthoc follow-up interview of participants residing in the lowlands of Ho Chi Minh City and the Mekong Delta. A total of 69 (90%) of 77 case-patients and 82 (32%) of 254 control-patients responded to the blinded posthoc follow-up phone interview. Participants who had lived or traveled to a high-risk region for >3 days were at significantly higher risk for talaromycosis (55/69 [81%] case-patients vs. 47/82 [57%] control-patients; OR 2.90 [95% CI 1.33–6.59]; p = 0.005).

## Discussion

This large case–control study investigated the behavioral, environmental, and geographic exposure risk factors for talaromycosis in susceptible persons living in a highly endemic region, Vietnam. Participants living within 100 meters of or having direct exposure to tropical plants (bamboo, sugar cane, or rice) or to farm animals (cattle, swine, or poultry) had 2-fold higher odds for talaromycosis. Our findings are consistent with the results of a previous smaller case–control study from Chiang Mai, Thailand (n = 180), which also found a 2-fold increase in the odds of talaromycosis with recent exposure to animals or plants (*18*). The strength of those associations is also similar across the 2 studies, despite the difference in study settings (urban vs. rural) and an 8-fold larger sample size in our study. Although direct exposure to soil was not identified as a risk factor in our study, soil is a known reservoir for other dimorphic fungal pathogens including *Coccidioides* spp., *Paracoccidioides* spp., *Histoplasma capsulatum*, and *Sporothrix schenckii* (*26**–**28*). Specifically, soil contaminated with animal droppings (bird or bat) is a known reservoir for *Histoplasma* spp., whereas soil mixed with decaying plant materials has been identified as a reservoir for *Sporothrix* spp. (*29*,*30*). Soil contaminated with farmed animal excreta or decaying tropical plant substrate is likely also favorable to *T. marneffei*’s growth, which supports the observed risk for talaromycosis in persons exposed to or living in proximity to animal and agricultural farming activities.

Direct contact with or consumption of bamboo rats was not associated with talaromycosis in our study, which is consistent with the previous case–control study from Thailand (*18*). Early efforts to isolate *T. marneffei* from the soil within bamboo rat burrows yielded some success (*14*,*31*,*32*). An environmental survey in Guangdong Province, China, demonstrated a higher prevalence of *T. marneffei* in soil collected from bamboo rat burrows (8.2% [15/184]) than in soil from sites not associated with bamboo rats (2.0% [1/50]) (*19*). *T. marneffei* was isolated in bamboo rat stool on the surface of the soil and around the bamboo roots found deep in the bamboo rat burrows (*19*). In Thailand, *T. marneffei* DNA was found in soil around a bat cave and elephant camp (*33*). However, the bamboo rat is the only known nonhuman host of *T. marneffei* (*16*,*19*)*. T. marneffei* is found in the liver, spleen, and lungs of healthy-appearing bamboo rats, suggesting that bamboo rats are asymptomatic carriers of *T. marneffei* and serve as a zoonotic reservoir (*19*). Bamboo rats construct extensive burrow systems for foraging among bamboo crops by digging 1–2 meters into the ground. Although their primary food source is bamboo roots and shoots, bamboo rats are known to feed on sugar cane and cassava roots. In our study, exposure to tropical plants was an independent risk factor for talaromycosis, possibly because of their association with the bamboo rat environment. By burrowing into the soil and feeding on a variety of tropical vegetation, bamboo rats likely enhance *T. marneffei* transmission. Soil disturbance, caused by bamboo rat foraging, heavy rainfall, or agricultural activity (e.g., tillage, plowing, livestock grazing), causes aerosolization of *T. marneffei* conidia, the infective form inhaled by bamboo rats and humans (*4*,*6*,*20*,*21*). The findings of our study suggest that exposure to soil associated with bamboo rats (and possibly other animals) and activities that disturb soil are the main drivers for the acquisition of talaromycosis, rather than direct zoonotic transmission.

Geographic mapping was very informative and demonstrated that the Central Highlands and the adjacent Southeast and South-Central Coast regions are high-risk regions for talaromycosis, compared with the lowlands of Ho Chi Minh City and the Mekong Delta regions in Vietnam. Assuming the referral patterns of patients with HIV were similar among regions, talaromycosis cases per 100,000 persons with HIV were 8–10 times higher in the Southeast and Central Highlands regions than in the Mekong Delta and were 2–3 times higher than in Ho Chi Minh City. Highland regions are the known natural habitat of bamboo rats, and previous studies outside of Vietnam have demonstrated that human talaromycosis follows the geographic distribution of bamboo rats (*16*,*17*). Further interrogation of participants from the lowlands of Ho Chi Minh City and Mekong Delta revealed that previous residence in or travel to highland regions increased the odds of talaromycosis 3-fold. Our study demonstrates an epidemiologic link between human talaromycosis and residence in or travel to highland regions in a highly endemic country. Further research is needed to determine whether localized geographic hotspots of talaromycosis exist within those highland regions. Our data can inform prevention strategies for talaromycosis, including patient education and the consideration of primary itraconazole prophylaxis in high-risk persons considering travel to or living in regional hotspots.

The first limitation of our study is that 31 persons with talaromycosis were screened but not enrolled in our study, largely because of discharge or death. Those participants might have exhibited different risk factors from those enrolled in the study, which is a potential source of selection bias; however, the number is very small (5.1%). Second, control-patients who had concurrent bacteremia could potentially have been misclassified because bacteria can outgrow *T. marneffei* in culture, leading to false-negative results. CD4 count was only available for 30% of participants, and therefore matching was performed on the basis of absolute lymphocyte count and WHO HIV disease staging, which are suboptimal measures of immune susceptibility. Some characteristics of case-patients and control-patients, such as median CD4 cell count, WHO HIV stage, and proportion of inpatients, differed significantly. However, our control population consisted of clinically relevant persons with advanced HIV disease (CD4 <200 cells/mL) and other opportunistic infections, which are representative of the population at risk for talaromycosis. Although interviewers were blinded to the classification of case and control, observer bias is possible, because 40%–70% of persons with talaromycosis display characteristic umbilicated skin lesions (*6*,*34*,*35*). However, that possibility was mitigated because interviewers were blinded to study hypotheses to minimize observer bias. The differential referral pattern between cases and controls in different regions might have skewed the geographic results, but that skewing was unavoidable because of limited diagnostic capacities outside of major referral centers in Vietnam. The number of cases recruited in Ho Chi Minh City and Hanoi was imbalanced because enrollment started earlier in Ho Chi Minh City, resulting in overrepresentation of residents from southern Vietnam. Finally, potential for recall bias is inherent to a case–control study design. Further studies are needed to uncover more specific geographic risk factors and risk behaviors and to establish the causal link between environmental exposure and the development of talaromycosis.

In conclusion, this study demonstrates an epidemiologic link between human talaromycosis and geospatial proximity or travel to highland regions in a hyperendemic country, thus informing disease education and prevention strategies. Results from this large case–control study validate the previous findings that disturbance of soil associated with tropical plants and farmed animals, such as through agricultural or construction activity, increases the risk for talaromycosis.

Appendix Additional information about multicenter case–control study of behavioral, environmental, and geographic risk factors for talaromycosis
