# Supplementary material for: Multicenter Case–Control Study of Behavioral, Environmental, and Geographic Risk Factors for Talaromycosis, Vietnam
Source: Emerg Infect Dis. 2025 Jul;31(7):1309–18. doi: 10.3201/eid3107.250143 (PMC12205468; doi:10.3201/eid3107.250143)
Supplement: Appendix — Additional information about multicenter case–control study of behavioral, environmental, and geographic risk factors for talaromycosis [file 25-0143-Techapp-s1.pdf]

Article DOI: https://doi.org/10.3201/eid3107.250143
EID cannot ensure accessibility for supplementary materials supplied by authors. Readers who have difficulty accessing supplementary content should contact the authors for assistance.

Multicenter Case–Control Study of Behavioral, Environmental, and Geographic Risk Factors for Talaromycosis, Vietnam

Appendix

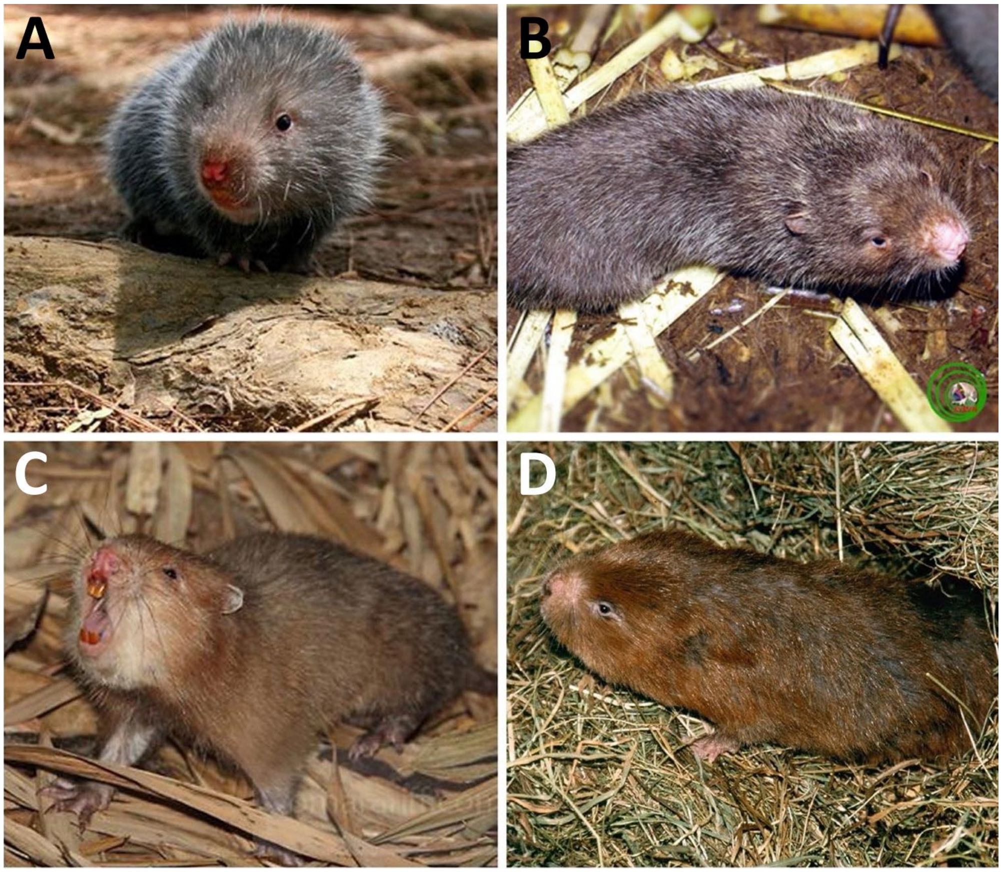

Appendix Figure 1. The 4 species of bamboo rat in Asia. A) *Rhizomys sinensis*, B) *Rhizomys pruinosus*, C) *Rhizomys sumatrensis*, D) *Cannomys badius*. Photo credits: Pham Quang Tung, http://dongbathoangda.com.vn (A); Phung My Trung, http://www.vncreatures.net (B); Omar Ariff, https://omarariff.photoshelter.com (C); Klaus Rudloff, http://www.biolib.cz (D). Permission was obtained from all photographers.

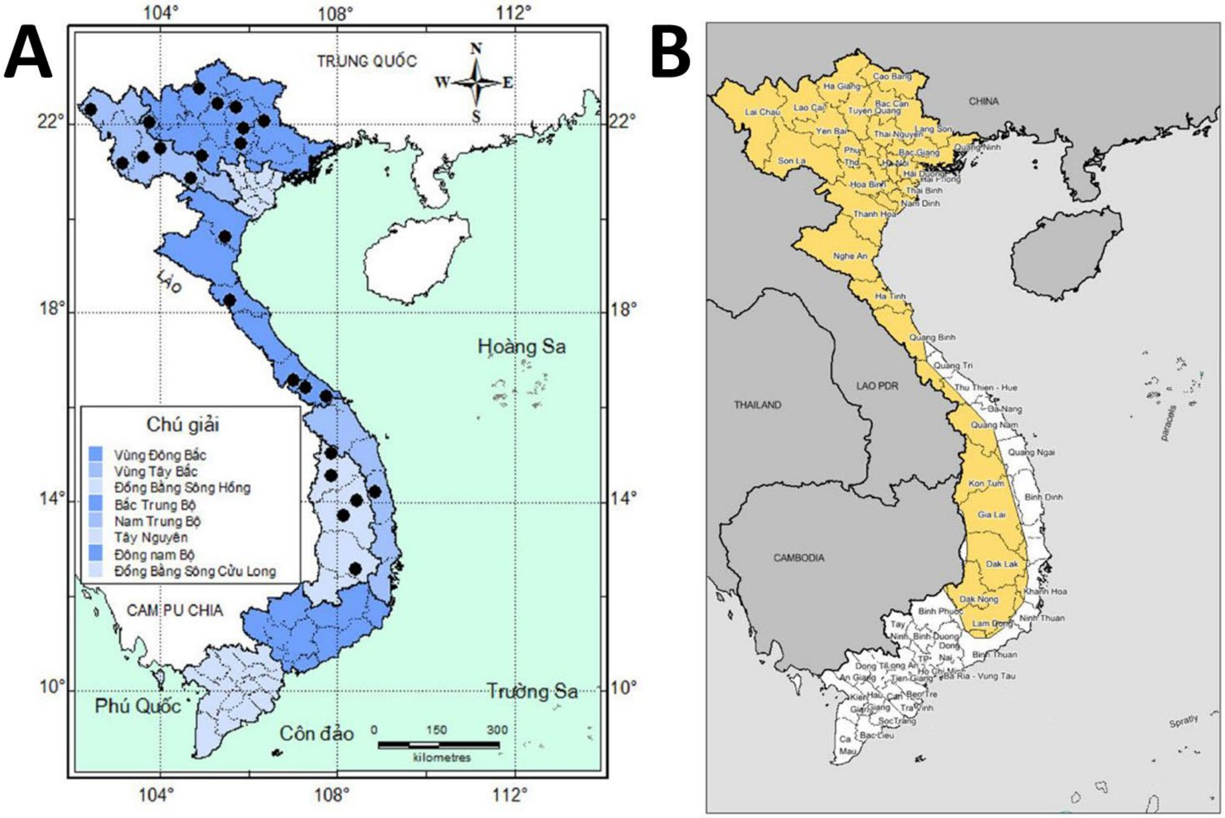

Appendix Figure 2. Maps of the distribution of *Rhizomys pruinosus* in Vietnam. A) Map created by Can D.N. et al. (*1*). Each black dot represents an occurrence record. Regions include Vùng Đông Bắc (Northeast region), Vùng Tây Bắc (Northwest region), Đồng Bằng Sông Hồng (Red River Delta), Bắc Trung Bộ (North Central Coast), Nam Trung Bộ (South Central Coast), Tây Nguyên (Central Highlands), Đông Nam Bộ (Southeast region) and Đồng Bằng Sông Cửu Long (Mekong River Delta). B) Map compiled in 2015 by Hoang Minh Duc on the International Union for Conservation of Nature Red List (http://www.iucnredlist.org/mammals). Both maps were used with permission from the original authors.

Reference

1. Can DN, Endo H, Son NT, Oshida T, Canh LX, Phuong DH, et al. Checklist of wild mammal species of Vietnam. Hanoi: Institute of Ecology and Biological Resources; 2008.
